# In Vivo Characterization of Dynein-Driven nanovectors Using *Drosophila* Oocytes

**DOI:** 10.1371/journal.pone.0082908

**Published:** 2013-12-12

**Authors:** Nadège Parassol, Céline Bienvenu, Cécile Boglio, Sébastien Fiorucci, Delphine Cerezo, Xiao-Min Yu, Guilhem Godeau, Jacques Greiner, Pierre Vierling, Stéphane Noselli, Christophe Di Giorgio, Véronique Van De Bor

**Affiliations:** 1 University of Nice Sophia-Antipolis, Institute of Biology Valrose (iBV), UMR 7277-CNRS, UMR 1091 INSERM, Nice, France; 2 Université de Nice Sophia-Antipolis, Institut de Chimie de Nice (ICN), UMR 7272-CNRS, Nice, France; University of Bern, Switzerland

## Abstract

Molecular motors transport various cargoes including vesicles, proteins and mRNAs, to distinct intracellular compartments. A significant challenge in the field of nanotechnology is to improve drug nuclear delivery by engineering nanocarriers transported by cytoskeletal motors. However, suitable *in vivo* models to assay transport and delivery efficiency remain very limited. Here, we develop a fast and genetically tractable assay to test the efficiency and dynamics of fluospheres (FS) using microinjection into *Drosophila* oocytes coupled with time-lapse microscopy. We designed dynein motor driven FS using a collection of dynein light chain 8 (LC8) peptide binding motifs as molecular linkers and characterized in real time the efficiency of the FS movement according to its linker’s sequence. Results show that the conserved LC8 binding motif allows fast perinuclear nanoparticle's accumulation in a microtubule and dynein dependent mechanism. These data reveal the *Drosophila* oocyte as a new valuable tool for the design of motor driven nanovectors.

## Introduction

Active transport in cells is largely driven by the kinesin and dynein motor families that move toward the plus and minus end of microtubules (MT), respectively [1]. Various pathogens have evolved mechanisms to hijack the cellular transport machinery allowing them to propagate efficiently. For example, many viruses are able to shuttle within the cell harnessing either dynein to reach the nucleus or kinesin to reach the cell membrane [2,3]. These strategies inspired nanotechnologists to design motor driven nanocarriers able to actively transport drugs or nucleic acids across the viscous cytoplasm toward the nucleus, allowing improvement of intracellular transport, bioactivity (gene expression), as well as reduction of cellular toxicity [4-6]. However, the lack of powerful experimental *in vivo* assays able to evaluate novel transport systems is holding back their development. Dynein is a large multi protein complex, containing a motor domain composed of two heavy chains (HC), and a cargo binding domain, made of intermediate chains (IC), light intermediate chains (LIC) and dimers of light chains including LC8 [7]. Dynein LC8 is essential and highly conserved with 90% of sequence identity between *Drosophila* and human [8]. Interestingly, *in vivo* and *in vitro* studies identified a short consensus LC8 binding sequence, (KXTQT) [9-12], found in a number of LC8 interacting proteins including viral proteins [13-15]. In *Drosophila*, the LC8 binding sequence is also present in proteins involved in dynein dependent mRNA transport such as Swallow (Swa) [16] and Egalitarian (Egl) [17]. Here, we developed a novel screening assay using live *Drosophila* oocyte microinjection combined with genetic, pharmacological and videomicroscopy. We designed dynein motor driven fluorospheres using a collection of dynein light chain 8 (LC8) peptide binding motifs as molecular linkers and demonstrate their ability to move toward the nucleus of *Drosophila* oocytes. We further showed that conserved LC8 binding motifs allow the rapid perinuclear accumulation of nanoparticles in a microtubule and dynein dependent mechanism. This new approach represents a valuable tool allowing the identification of new motor-cargoes biolinkers and the genetic characterization of their intracellular transport efficiency.

## Results and Discussion

**Figure 1:**
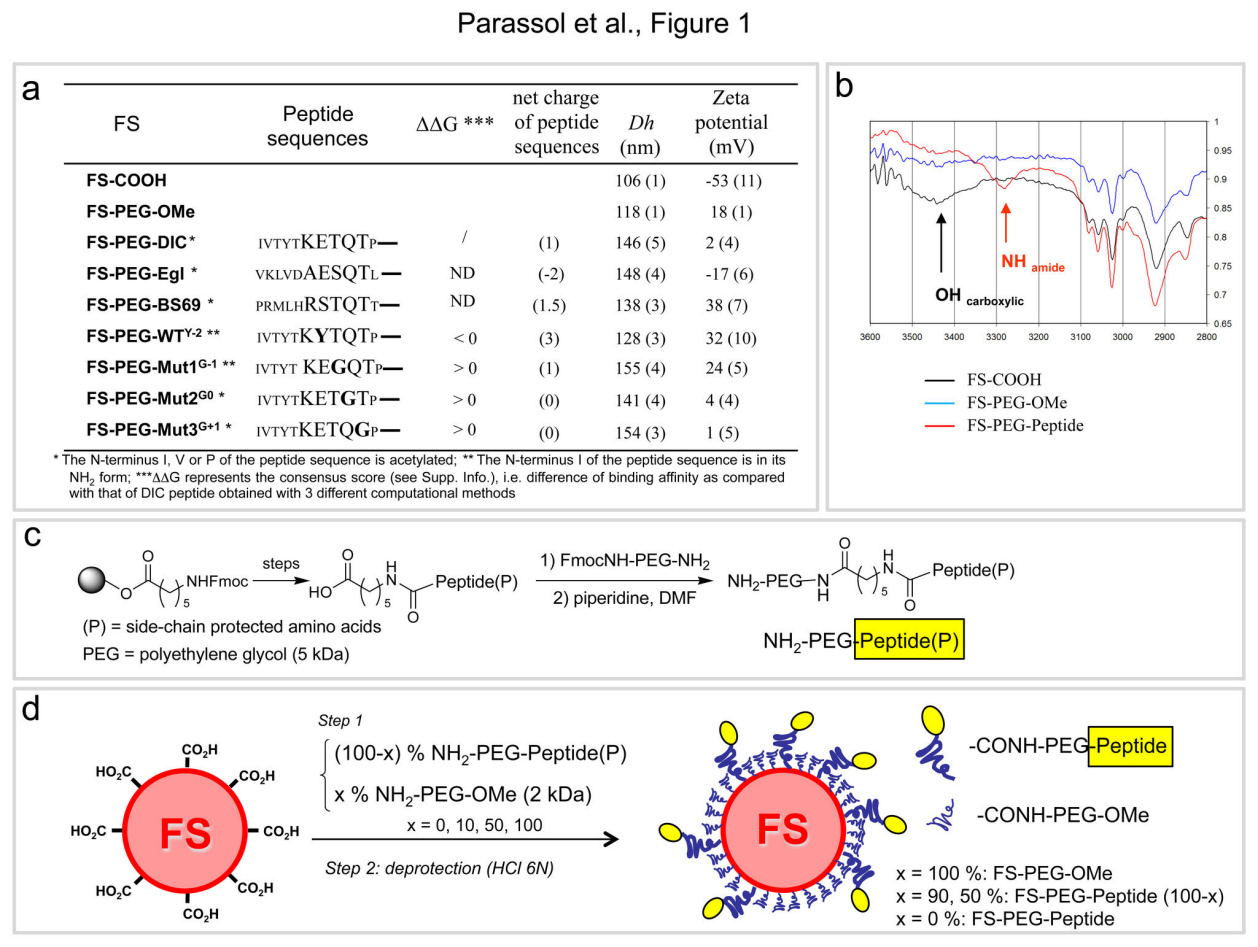
Preparation and characterization of functionalized fluospheres with various PEG-Peptide conjugates. (a) Table showing the nomenclature of the various FSs, the corresponding peptide sequence, the binding affinity difference (ΔΔG consensus score) of the peptide for dynein as compared with that of DIC peptide according to three computational methods and the residual charge of the peptide at physiologic pH, the hydrodynamic diameter, Dh (SD) and zeta potential (SD). (b) IR spectrum (part corresponding to the X-H vibration) of the peptide-functionalized FSs as compared to that of the starting carboxylated FSs and FS-PEG-OMe: the presence of amide N-H vibrations centered at 3280-3290 cm^-1^ confirms the conjugation of the peptide onto the FSs. (c) Solid phase synthesis of protected PEG-Peptide(P) conjugates. (d) Functionalization of the carboxylated FSs by the PEG-Peptide conjugates.

**Figure 2:**
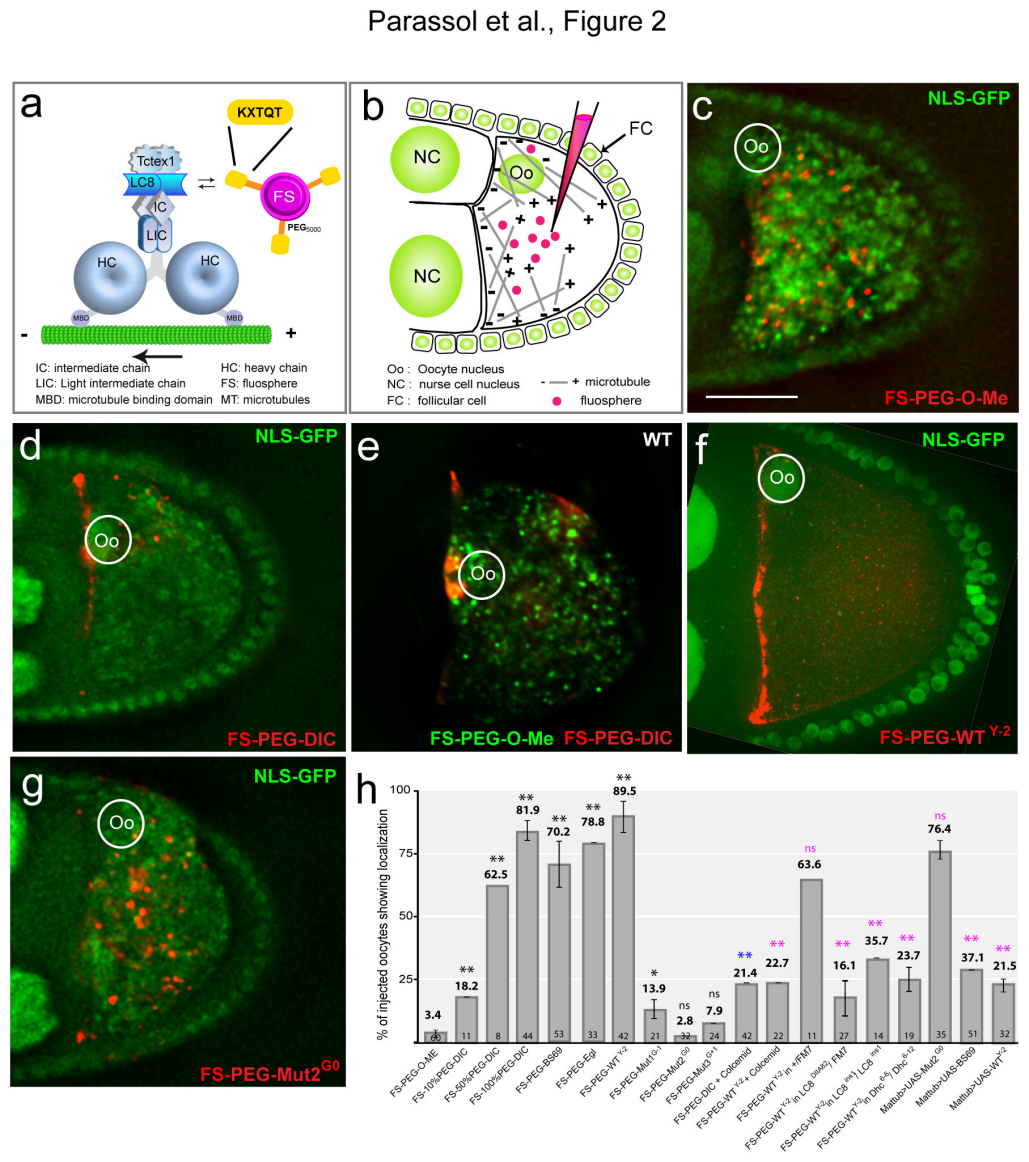
Conserved LC8 binding motifs can be used as molecular linkers between cargoes and dynein. (a) Functionalized FSs and dynein complex putative interaction scheme. (b) Injection assay scheme. In the oocytes MTs nucleate from the antero-lateral cortex and show an antero-posterior gradient. (c) FS-PEG-OMe spread randomly when injected into living nls-GFP oocyte (nls-GFP marks nuclei). In this and all subsequent figures the dorsal is to the top, the posterior to the right and (Oo) indicates the oocyte nucleus. Oocytes are shown 45 min after injection. Scale bar equals 50 μm. (d) Injected FS-PEG-WT concentrate at MT minus ends. (e) green FS(488/685)-PEG-OMe and red FS-(543/620)-PEG-WT co-injection. Only the red FS(543/620)-PEG-WT localize to the MT minus ends. (f) FS-PEG-WT^Y-2^ injection shows localization at the anterior pole. (g) FS-PEG-Mut2^G0^ shows no specific localization. (h) Graph representing the percentage of injected oocytes showing localization. The numbers in the bars represent the number of injected oocytes. Error bars represent +/- SEM. The mean and the SEM were calculated on independent experiments (n ≥ 3). *, p<0.05; **, p<0.005, (ns) non significant; in black, Chi-2 test of the different conditions versus FS-PEG-O-Me; in blue, Chi-2 test of FS-PEG-DIC + colcemid versus FS-100%PEG-DIC; in pink, Chi-2 test of the different conditions versus FS-PEG-WT^Y-2^ (5% critical value, 1 degree of freedom).

In order to assess the ability of LC8 binding peptides to behave as molecular linkers with the dynein complex, we functionalized 100 nm polystyrene carboxylated fluospheres (FS) with various peptides and tested their capacity to travel in the cytoplasm after injection into *Drosophila* oocytes (Figure 1a-1d; Figures 2a, 2b). Peptides’ sequences and length were determined by molecular modeling based on peptide/dynein X-ray structures available in the Protein Data Base and using various scoring methods (Material S1, Figure S1 and Table S1). To externalize the peptides from the FS's surface and favor LC8 interaction, peptides were conjugated at their C-terminus (to respect the physiological orientation) to the surface through 5kDa hydrophilic polyethylene glycol (PEG) spacer (Figure 1c, 1d; see Methods). *Drosophila* oocytes are large cells harboring a polarized cytoskeletal network that is well characterized [18,19]. Moreover, it is established that mRNA cargoes essential for oocyte development are transported by dynein toward the MT minus ends that concentrate near the nucleus [20-27] (Figure 2b). After injection, FS's behavior was recorded using time-lapse microscopy at a rate of 1 image every 5 min.

First, injected control FSs tagged with PEG only (FS-PEG-OMe) (see Methods) spread randomly in the oocyte cytoplasm and showed no specific accumulation (Figure 2c, 2h) (Movie S1). FS's injection is non-toxic for the cell as they continue to develop like uninjected oocytes (not shown). Docking calculations showed that a peptide of 11 amino acids containing the DIC consensus sequence IVTYT**KETQT**P (DIC) could be considered as being among the best LC8 ligands. We tested LC8 binding motifs identified in three known LC8 binding proteins: dynein intermediate chain (DIC), adenovirus-associated protein BS69 [15] and Egl [17]. FSs carrying these peptides (FS-PEG-DIC, FS-PEG-BS69 or FS-PEG-Egl) all behaved similarly: they concentrated toward the MT minus ends within minutes after injection, and accumulated around the nucleus over time in 70 to 82% of injected oocytes, the DIC peptide showing the best score (Figure 2d, 2h) (Movie S2). To rule out that localization was due to the injection protocol, we co-injected a mix (1:1) of control green-labeled FSs (FS-PEG-OMe) and red-labeled FSs functionalized with the DIC peptide. The FS-PEG-DIC concentrated around the nucleus whereas the FS-PEG-OMe were evenly distributed (Figure 2e) (Movie S3) confirming that localization is specific and dependent on the peptide. To test further the specificity of the approach and the effect of the amount of peptide per FS on its dynein transport, we progressively increased the percentage of PEG-DIC peptides around the FSs. The surface of the FSs was saturated with either a mixture of PEG-peptide/PEG2000-OMe conjugates (10/90 mol %, 50/50 mol%) or 100% PEG-peptide. Interestingly, increasing the amount of peptides from 10% to 100% dramatically improved the FS's perinuclear localization (Figure 2h). We therefore concluded that localization is dependent on the amount of bound peptides and therefore tagged the spheres with 100% of PEG-peptide conjugates for the rest of the study. In this condition, the close proximity of peptides at the surface may allow dimeric forms of peptide showing higher affinity for LC8 chains than monomers [13]. Altogether, these data validate our assay and reveal that heterologous dynein LC8 binding motifs are functional in *Drosophila* and can be used as universal molecular linkers. 

To challenge our approach and modeling, we then tested the FSs tagged with the peptide sequence WT^Y-2^ predicted to have slightly improved interaction with LC8 homodimer (Figure 1a). As expected, the FS-PEG-WT^Y-2^ localized to the MT minus ends more efficiently in ~90% of injected oocytes (~82% for FS-PEG-DIC) (Figure 1f, 1h) (Movie S4). We also assayed the localization ability of FSs associated with peptide containing a TQT core motif mutant sequence Mut1^G-1^ (T-G), Mut2^G0^ (Q-G) and Mut3^G+1^ (T-G) predicted to have a low binding affinity for LC8 (Figure 1a). As expected, these FSs (FS-PEG-Mut1^G-1^, FS-PEG-Mut2^G0^ and FS-PEG-Mut3^G+1^) failed to localize and were instead evenly distributed within the oocyte (Figure 2g, 2h) (Movie S5). Hence, the localization capability of FSs correlates with our calculation of peptides affinity for the LC8 dimers. Altogether, these results show that injection of traceable cargoes in *Drosophila* oocytes is a fast, specific and highly sensitive method to characterize nanovector's intracellular behavior. 

**Figure 3:**
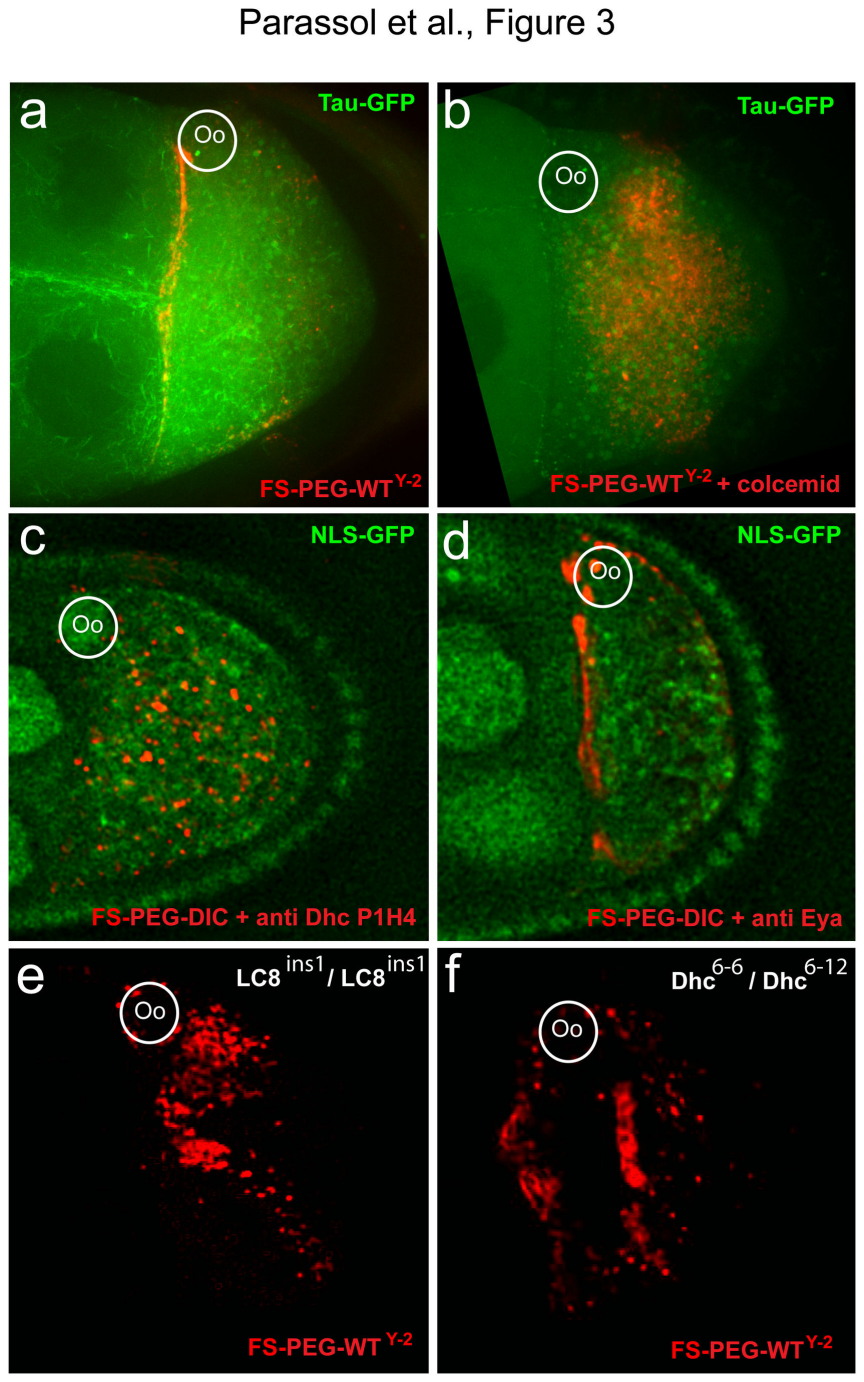
Transport of functionalized cargoes is MT and dynein dependent. (a, b) Tau-GFP oocyte. (a) FS-PEG-WT^Y-2^ shows specific localization to the anterior pole. (b) FS-PEG-WT^Y-2^ co-injected with colcemid fail to localize. (c) FS-PEG-WT co-injected with an anti-Dhc (P1H4) antibody fail to localize. (d) Co-injection of a control anti-Eya antibody does not affect FS-PEG-WT localization. (e) Localization of FS-PEG-WT^Y-2^ into an LC8 ^ins1^/*LC8* ^ins1^ hypomorphic mutant is perturbed. (f) Localization of FS-PEG-WT^Y-2^ into a *Dhc* ^6-6^/*Dhc* ^6-12^ hypomorphic mutant is affected. Oocytes are shown 45 min after injection.

We next asked whether MTs were required for FS's localization. FS-PEG-WT^Y-2^ beads were injected into nod-LacZ oocytes in order to visualize MT's minus ends. Interestingly, MT's minus ends and the FSs showed colocalization (Figure S2a, S2b). Tau-GFP oocytes were then co-injected with the FSs and colcemid, a drug preventing MT polymerization. The toxin strongly affects MT's organization and alters perinuclear localization of FS-PEG-DIC and FS-PEG-WT^Y-2^ beads (Figure 3a, 3b, 2h) (Movie S6). To test the requirement for dynein, we co-injected the FSs with an antibody against Dhc, known to prevent dynein dependent transport [28], and found it severely affects FS's localization (Figures 3c, 2h) (Movie S7). In contrast, injection of a control antibody had no effect (Figure 3d, 2h compared to Figure 2d). Finally, FSs were injected in hypomorphic mutant conditions for *LC8* (*Dlc* ^ins^/*Dlc* ^ins^) or *Dhc* (*Dhc* ^6‑6^/*Dhc* ^6‑12^). FS's localization is strongly affected in both mutant conditions, with 60% of injected oocytes showing random distribution of the FSs (Figure 3e, 3f, 2h). These results show that localization is microtubule and dynein dependent and requires the LC8 and heavy chain subunits.

**Figure 4:**
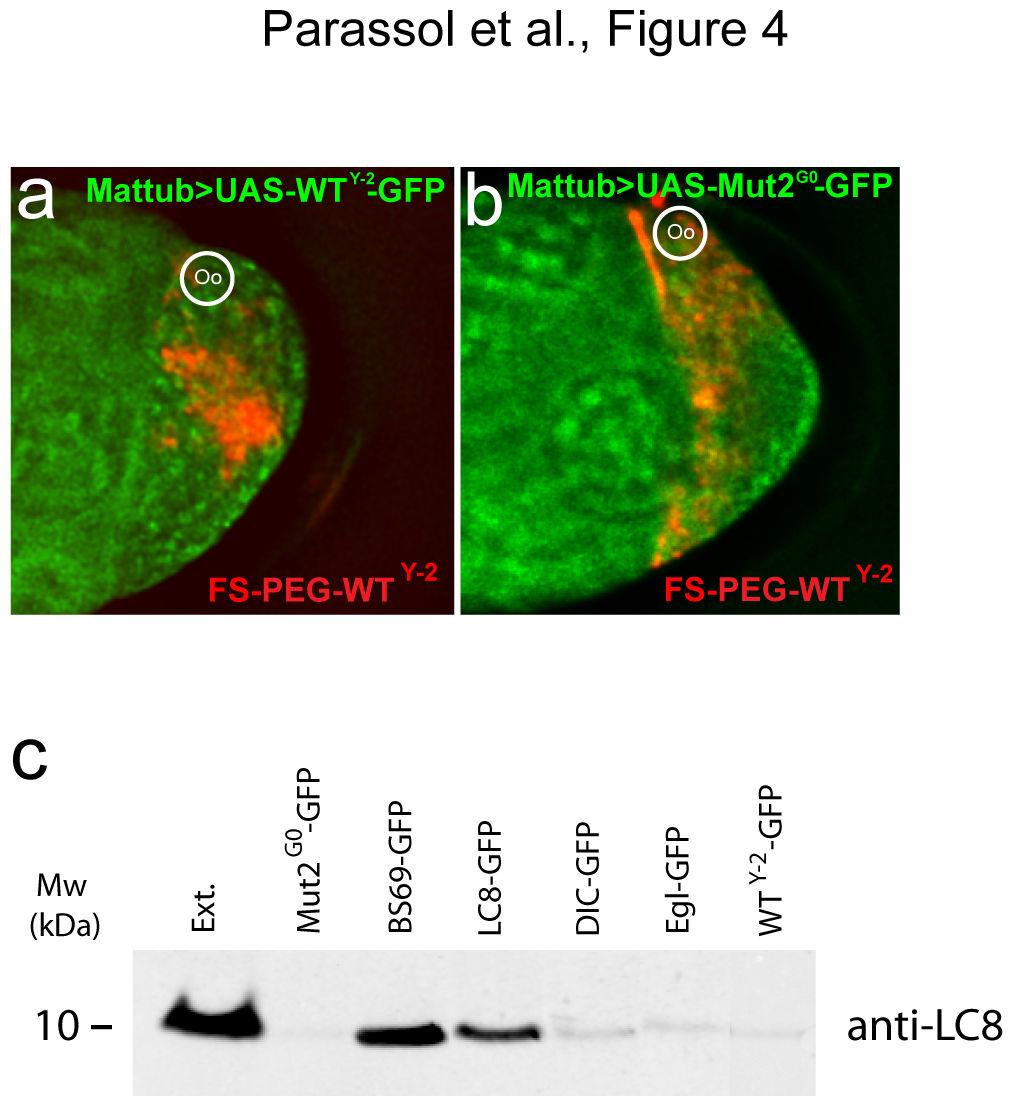
LC8 binding peptides interact with endogenous LC8. (a) Overexpression of WT^Y-2^ -GFP tagged peptide prevents the localization of FS-PEG-WT^Y-2^. (b) Overexpression of Mut2^G0^-GFP tagged peptide does not affect FS-PEG-WT^Y-2^ localization. Oocytes are shown 45 min after injection. (c) Immunoblot of oocyte extracts expressing GFP tagged peptides (Mut2^G0^-GFP, BS69-GFP, DIC-GFP, WT^Y-2^-GFP Egl-GFP) or full length LC8 (LC8-GFP). Immunoprecipitation was performed using an anti-GFP antibody and western blot using an anti-LC8. Full length LC8 as well as LC8 binding peptides except for Mut2^G0^ associate with endogenous LC8 under physiological salt conditions.

To characterize *in vivo* the interaction between LC8 and the LC8 binding peptides, we engineered transgenic fly lines expressing DIC, WT^Y-2^ or BS69 peptides fused to GFP. Expression of the transgenes in the oocyte led to a strong cytoplasmic GFP signal. Strikingly, localization of FS-PEG-WT^Y-2^ is strongly decreased in oocytes expressing DIC-GFP, BS69-GFP and WT^Y-2^-GFP. In contrast, FS-PEG-WT^Y-2^ efficiently localize in Mut2^G0^-GFP expressing flies (Figure 4a, 4b, 2h) (movie S8). These results suggest that functionalized FSs and GFP-tagged peptides compete for LC8 binding. To further characterize the biochemical interaction, oocytes overexpressing DIC-GFP, BS69-GFP, WT^Y-2^-GFP, Egl-GFP, LC8-GFP or Mut2^G0^-GFP tagged peptides were lysed and GFP was immunoprecipitated. Western blot analysis revealed that endogenous LC8 co-immunoprecipitates with DIC-GFP, BS69-GFP, WT^Y-2^-GFP, Egl-GFP, LC8-GFP but not with Mut2^G0^-GFP peptides (Figure 4c). These data indicate that LC8 binding peptides interact directly or in a complex with endogenous LC8 and allow transport toward the nucleus by the dynein complex.

**Figure 5:**
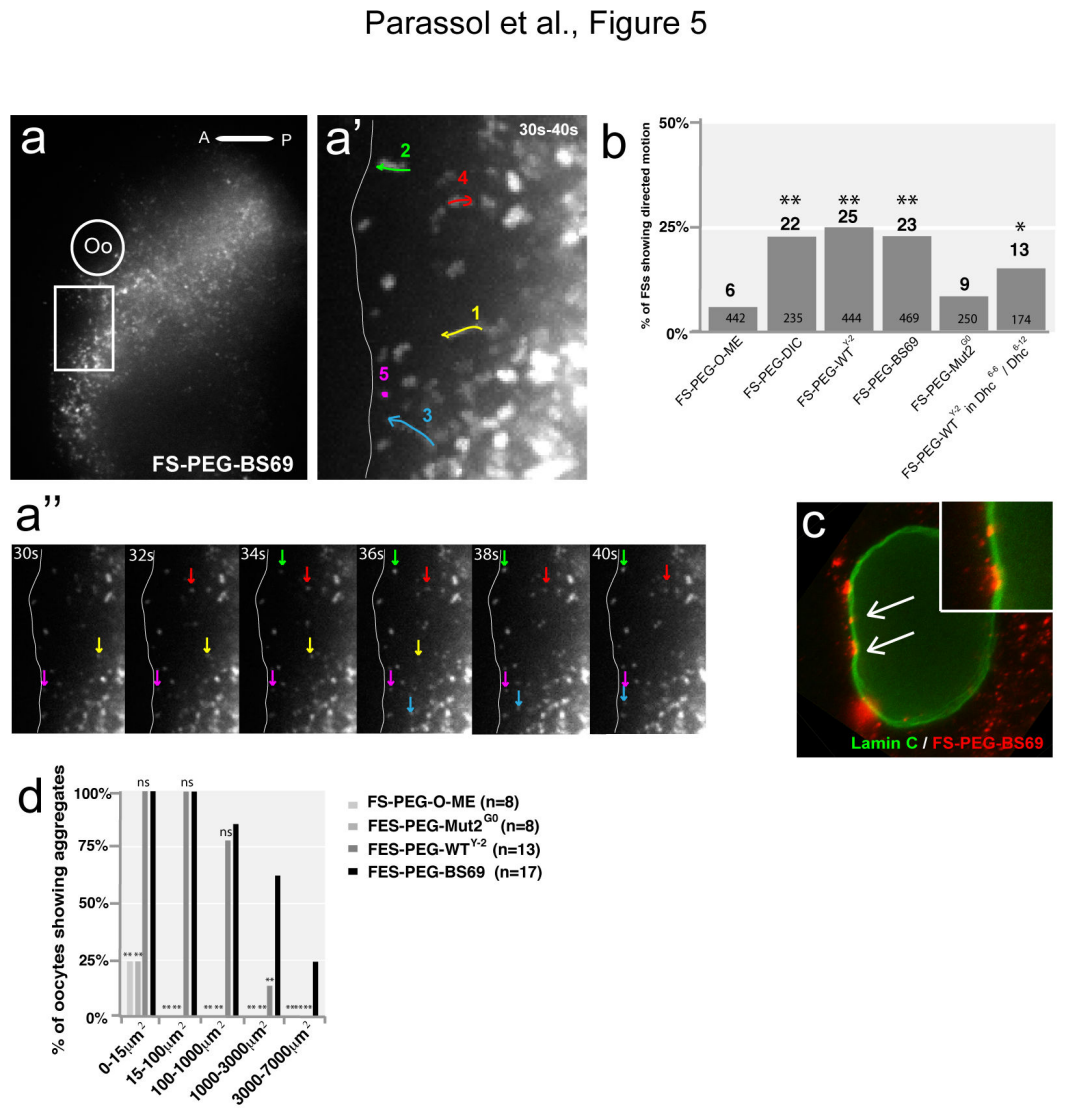
Intracellular movement of functionalized FSs reveals active and polarized transport. (a) FS-PEG-BS69 injection into nls-GFP oocyte, 5 min post injection. White box highlights the region shown in a’, (Oo) indicates the oocyte nucleus, (A) anterior, (P) posterior. (a’) 20 time points (30 seconds (30 s) to 40 seconds (40 s)) projection from a 1 min movie taken 5 min after injection (2 frames/s). Arrows 1, 2 and 3 follow the path of actively transported particles; arrows 4 and 5 follow particles showing diffusion and static behavior, respectively. Movement characterization was made by MSD analysis. White line indicates the anterior boundary of the oocyte. (a’’) Series of 6 frames extracted from the movie. Yellow, green, blue, red and pink arrows show the movement of particles 1, 2, 3, 4, 5, respectively. Oocyte is on the left of the frame. (b) Graph showing the percentage of actively transported particles determined by MSD calculation. The numbers in the bars represent the total number of analyzed fluospheres for each condition. *, p<0.05; **, p<0.005; Chi-2 test of the different conditions versus FS-PEG-O-Me (5% critical value, 1 degree of freedom). (c) 30min after injection, FS-PEG-BS69 form aggregates around the oocyte nucleus. Some aggregates are closely associated to the nuclear envelope (arrows). (d) Graph showing the percentage of injected and fixed oocyte (45 min after injection) showing FSs aggregates (0 μm^2^ <maximum size <7000 μm^2^) around the nucleus. The numbers represent the total number of analyzed oocyte for each condition. *, p<0.05; **, p<0.005; Chi-2 test of the different conditions versus FS-PEG-BS69 (5% critical value, 1 degree of freedom).

To characterize the dynamics of actively transported FSs, we captured their intracellular behavior after injection using fast (2 images/sec) and high resolution time lapse imaging. Movies were taken 5 to 10 min post injection near the nucleus and during 1 min. The mean square displacement (MSD) was calculated for each individual particle and plotted versus Δt. FS's movements were classified into three groups of motility based on theoretical previous studies [29–31]: static (particle show little to no motion; MSD curve is a straight line and α ≤ 0.1 ), Diffusion (MSD increases linearly with the time intervals and 0.1< α < 1.2) or directed motion (MSD plot gives a parabolic curve and α ≥ 1.2) (Figure 5a, 5a’,5a’’) (movie S9). Interestingly, only 6% of injected FS-PEG-OMe show directed motion compared to approximately 22% for FS-PEG-WT, 23% FS-PEG-BS69 and 25% for FS-PEG-WT^Y-2^ with average speeds of 0.4 μm/s, consistent with active transport (Figure 5b). A majority of FS-PEG-WT (66%), FS-PEG-WT^Y-2^ (61%) and FS-PEG-BS69 (68%) move anteriorly toward the MT minus ends. In Dhc hypomorphic mutant, the percentage of FS-PEG-WT^Y-2^ showing directed motion is decreased, which could explain the decreased localization index observed (Figures 5b, 3f). Finally, we noticed that FSs functionalized with BS69 (65%, n= 17) or WT^Y-2^ (15%, n= 13) peptides but not with Mut2^G0^ (0%, n=8) accumulate around the nucleus forming aggregates (size > 1000 μm^2^) adjacent to the nuclear envelope suggesting that LC8 binding peptides could facilitate nuclear delivery (Figure 5c) (movie S10). These findings indicate that LC8 binding may not only couple cargoes to the dynein complex but also promote their delivery to the nucleus. 

The LC8 subunit is highly conserved among species and ubiquitously expressed. Null mutations in the *Drosophila* gene are lethal showing that it has essential functions [32]. However, the role of LC8 in dynein driven cargoes transport is still debated and several attempt have been made to elucidate its function [5,6,33]. LC8 may promote the assembly of the complex through its dimerization potential and/or target the complex to specific cargoes.

Here we show using real time videomicroscopy that the LC8 binding motif is able to link cargoes to the dynein complex *in vivo*. In addition, our results indicate that LC8 might also promote nuclear delivery in *Drosophila*, a feature that may be shared between *Drosophila* and other species [34]. Structural studies have demonstrated that LC8 works as a dimer able to bind dimer of DIC fragment within the motor complex. To act as a cargo adaptor, one of the LC8 monomer would have to interact with the IC and the other with the FSs peptides. However, the nature of the LC8 interaction (dimer) with DIC would make such a scenario very unlikely. Interestingly, there are number of evidences that LC8 subunits can form tertiary and quaternary structures. In yeast, five Dyn2 homodimers (the ortholog of LC8) interact to stabilise and promote the dimerization of Nup 159, a component of the nuclear pore complex [34]. Moreover, it was demonstrated *in vitro* that rat LC8 subunits can form “dimer of dimer” [10] and tetramer of human LC8 were observed by gel permeation chromatography [5]. Finally, based on x-ray crystallography, it has been hypothesized that LC8 could function as a hub protein promoting dimerization and multivalency of its partners [15,35]. Based on these data, one attractive model would be that LC8 could play the role of cargo adaptor through transient tetramerisation. In this model, one LC8 homodimer could bind to the motor complex through the DIC subunits, the second homodimer to the FSs peptide. 

In this study, we found that G0 mutant peptide, consistently failed to localize fluospheres and to bind LC8. Interestingly, while BS69, DIC, Egl and WT^Y-2^ allow comparable fluospheres’ localization, we observed an enhanced binding of BS69 to LC8 compared with DIC, Egl or WT^Y-2^. This result is not due to major variation of peptide level since transgenes expressions appear similar on immunoblot (data not shown). The BS69 sequence is of viral origin and it has already been shown that viruses are able to efficiently hijack the cellular transport machinery [2,3]. We thus interpreted that BS69 interaction with LC8 is more stable preventing dissociation of the complex, whereas DIC, Egl and WT^Y-2^ affinities may induce transitory interaction and frequent dissociation of the complex. While nuclear accumulation is apparent with all peptides it could generate differences in pulldown efficiency. Finally, the tight binding of BS69 to LC8 might also be responsible for the large FS-PEG-BS69 aggregates observed around the nucleus.

In conclusion, *Drosophila* oocytes provide a suitable and powerful system to characterize and develop nanovectors able to transport therapeutic cargoes to specific intracellular compartments including the nucleus. Direct injection in the cytoplasm of the large oocyte was shown to allow for fast, efficient, non-cytotoxic testing of heterologous materials circumventing the cell entrance step. As shown in this study, *Drosophila* oocytes represent a unique system combining genetic, pharmacology and real time videomicroscopy analysis in a single package. Our approach can be adapted to test and improve various kinds of carriers including gold and magnetic particles combined with a large spectrum of motor biolinkers.

## Methods

### Fly strains

Flies were raised on standard cornmeal-agar medium at 25°C. The following strains were used: nlsGFP, tauGFP, ctp-GFP, Mat-tub-gal4 were from the Bloomington stock center (BL# 7062). Coding sequence for GFP tagged peptides were cloned in pUASp vector using KpnI-NotI cloning site. UASp-GFP-LC8, UASp-Egl-GFP, UASp- Mut2^G0^-GFP, UASp-WT^Y-2^-GFP, UASp –BS69-GFP were cloned KpnI, NotI. LC8 ^DIIA82^ and LC8^ins1^ mutants were from K. Ray’s lab. Dhc64c^6–6^ , Dhc64c^6-12^ were from T. Hays’ lab.

### Injection assay, MT drug treatment and Immunohistochemistry

For injection, ovaries were dissected in halocarbon oil [26]. Injections were performed using a femtojet (Eppendorf). For immunohistochemistry, ovaries were prepared as described in [26] and mounted in Vectashield-DAPI (Vector). Primary antibodies were: anti-P1Η (provided by T. Hays, 1:2 dilution for inection), anti-Eya (DSHB, 1:2 dilution for injection), anti-ddlc1 (provided by K. Ray’s lab), anti-LC8 (Abcam EP1660Y, ab 51603, WB dilution 1:2000), anti- LaminC (DSHB 1:100), anti-GFP (Antibodies Inc, 2 μl for IP, WB 1:500) anti-GFP (Life technologies, WB 1:1000, 2 μl for IP). MT alteration was induced by colcemid injection (100 μg/ml) (Sigma). 

### Western blot, immunoprecipitation

Westerns blots were performed on protein extracts from pools of three different strains for a same construct. Extracts protein concentrations’ were evaluated using Bio-Rad protein assay method based on the Bradford assay (Bio-Rad). We used the same total amount of protein per extract for each experiment (approximately 750 μg). IP were performed overnight at 4°C. A mix 1:1 of protein A sepharose: protein G sepharose was used (2 hours at RT). Beads were washed 3 times 10 min with the assay buffer (50 mM Tris pH 7.5; 150 mM NaCl; 1 mM MgCl_2_; 1% triton X100) before running the blots. 

### Functionalization of fluospheres with the PEG-peptide conjugates

The NH_2_-PEG-Peptide(P) conjugate carrying the protected DIC, Egl, BS69, WT^Y-2^, Mut1^G-1^, Mut2^G0^, or Mut3^G+1^ peptide sequence (0.002 mmol; see supplementary information for their synthesis and characterization) was dissolved into a mixture of MES (50 mM, pH 6, 200 μL) and DMF (200 μL). 543/620 or 488/645 carboxylated fluospheres from Molecular Probes (100 nm, 150 μL, 1.8x10^+13^ particles, 0.01 mmol) were then added. After 15 min of stirring, EDC (4 mg) was added and the mixture was incubated for 18 h. Remaining unreacted carboxylated functions were capped with glycine (100 mM) for 30 min. The functionalized fluospheres FS-PEG-Peptide (P) were collected after 5 cycles of centrifugation/resuspension (sonication) (14000 rpm, 30 min) with ultrapure water (1 mL). After the last cycle, supernatant was discarded and freshly prepared HCl 6N solution (400 μL) was added to FS-PEG-Peptide(P) for the removal of the various aminoacid side chain protecting groups. The mixture was vortexed for 96-120 h. Deprotected FS-PEG-Peptide beads were then purified through 10 cycles of centrifugation/resuspension (sonication) with ultrapure water (1.5 mL) till conductivity reaches ultrapure water one’s. As for the FSs carrying both the PEG-Peptide and PEG-OMe, they were prepared according to the same two-step procedure described above but starting with a 90/10 or a 50/50 mol% mixture of NH_2_-PEG-OMe (2 kDa) and NH_2_-PEG-Peptide (P). Conjugation of the peptides onto the FS's surface was confirmed by IR spectroscopy, which indicated the presence of NH vibrations (Figure 1). Hydrodynamic diameter and zeta potential of the FSs were further determined by dynamic light scattering and mixed-mode measurement phase analysis light scattering using a Zetasizer nano (Malvern Instruments, Paris, France). Basically, 20 µL of FSs were added to 680 µL of freshly prepared HEPES buffer (pH 7.2, filtrated over 0.22 µm). Smooth correlation curves associated with narrow sizes in the 130-160 nm range and low polydisperse indexes (from 0.06 to 0.26) ensure the full completion of the deprotection process. Conversely, remaining peptide protecting groups onto functionalized FSs showed erratic results with large sizes and polydisperse indexes (aggregates) and distorted correlation curves (see supplementary information). Furthermore ζ potentials fit quite nicely to the total charge of peptides when their side chains are free (see supplementary Methods).

### Imaging, deconvolution and statistical method

Imaging was performed on a confocal microscope Zeiss LSM 510 or on a widefield DeltaVision microscope (Applied Precision, DV 3.0 system, IX70 Olympus microscope, and CH350 camera) and deconvolved using SoftWorx software. 100x, fast imaging was performed on a Spinning disk microscope (OlympusAndor technology). Particle tracking was made manually using Image J. The speed of particles was calculated in micrometers per second according to the distance travelled in each time interval. The mean square displacement (MSD) was calculated according to the published formula [29,36] for each individual particle and plotted versus Δt. To determine the αcoefficient, the slope of the curve was determined on a loglog scale. FS's movement were classified into three groups of motility based on theoretical previous studies [29–31,37,38]: static (MSD curve is a straight line and α ≤ 0.1), diffusion (MSD increases linearly with the time intervals and 0.1< α < 1.2) or directed motion (MSD plot gives a parabolic curve and α ≥ 1.2). Curve fitting function of Excel was used for calculation. Statistical significance of fluospheres’ localization and movement was evaluated using a Chi^2^ test (5% critical value, one degree of freedom). For aggregates quantifications, oocytes have been injected, fixed and collected 45 min after injection. Oocytes were stained for Lamin C in order to visualise the nuclear envelope together with the fluospheres. Statistical significance was assessed using a Chi-2 test of FSPEGBS69 versus FSPEGPeptide (5% critical value, 1 degree of liberty) *, p<0.05; **, p<0.005. Aggregates characterization was made using Image J, the data presented correspond to the maximum surface of each object obtained using 3D object counter. 

## Supporting Information

Figure S1 **DIC peptide-protein complex structure.** Peptide ribbon is drawn in cyan and dynein light chain is in gray.
(DOCX)

Figure S2 **Functionalized FSs localize at the minus ends of MTs.** (a) βGal staining of a Nod-LacZ oocyte showing the minus ends of MTs. (b) Localized FS-PEG-WT^Y-2^ colocalized with βgal staining (arrow head).
(TIF)

Materials S1 **Molecular Modeling of Dynein peptides.**
(DOCX)

Movie S1 **FS-PEG-OMe move randomly through the oocyte cytoplasm.** FS-PEG-OMe injection in a NLS-GFP oocyte. Movie starts 30 min +/- 5 mins after injection in this and all subsequent movies unless otherwise mentioned. The frame rate is 1 image every 5 min in this movie and all subsequent movies unless otherwise mentioned.
(AVI)

Movie S2 **FS-PEG-DIC localize specifically close to the oocyte nucleus.** FS-PEG-DIC injection in a NLS-GFP oocyte.
(AVI)

Movie S3 **Co injected FS-PEG-DIC (red) and FS-PEG-OMe (green) show different behaviour in the oocyte cytoplasm.** FS-PEG-DIC (red) and FS-PEG-OMe (green) co-injection in a W118 oocyte.
(AVI)

Movie S4 **FS-PEG-WT^Y-2^ localize specifically close to the oocyte nucleus.** FS-PEG-WT^Y-2^ injection into a nls-GFP oocyte.
(AVI)

Movie S5 **FS-PEG-Mut3^G+1^ move randomly through the oocyte cytoplasm.** FS-PEG-Mut3^G+1^ injection in a nls-GFP oocyte.
(AVI)

Movie S6 **Colcemid inhibits FS-PEG-DIC specific localization.** Colcemid and FS-PEG-DIC co-injection in a Tau-GFP oocyte. Movie starts 20 min after injection.
(AVI)

Movie S7 **P1H4 inhibits FS-PEG-DIC specific localization.** P1H4 and FS-PEG-DIC co-injection in a Tau-GFP oocyte.
(AVI)

Movie S8 **FS-PEG-WT^Y-2^ compete for LC8 binding.** Left panel, FS-PEG-WT^Y-2^ localization is strongly decreased in oocytes expressing WT^Y-2^-GFP. Right panel, FS-PEG-WT^Y-2^ efficiently localize in Mut2^G0^-GFP expressing oocyte.
(AVI)

Movie S9 **FS-PEG-BS69 movement isdirected toward the anterior.** FS-PEG-BS69 move anteriorly. Colored lines follow FS showing directed motion toward the MT minus ends. The frame rate is 2 images/s. Movie starts 5 min after injection.
(AVI)

Movie S10 **FS-PEG-BS69 form aggregates embedded into the nuclear envelope.** 3D reconstruction and rotation around the nucleus of an oocyte injected with FS-PEG-BS69 and stained for Lamin C (blue) 45 min after injection. This oocyte is the same as in Figure 5d.
(AVI)

Table S1 **Binding free energy differences of mutant sequences related to DIC peptide according to various computational methods.**
(DOCX)
