# Supplementary figures and images for: In Vivo Characterization of Dynein-Driven nanovectors Using Drosophila Oocytes
Source: PLoS One. 2013 Dec 12;8(12):e82908. doi: 10.1371/journal.pone.0082908 (PMC3861458; doi:10.1371/journal.pone.0082908)

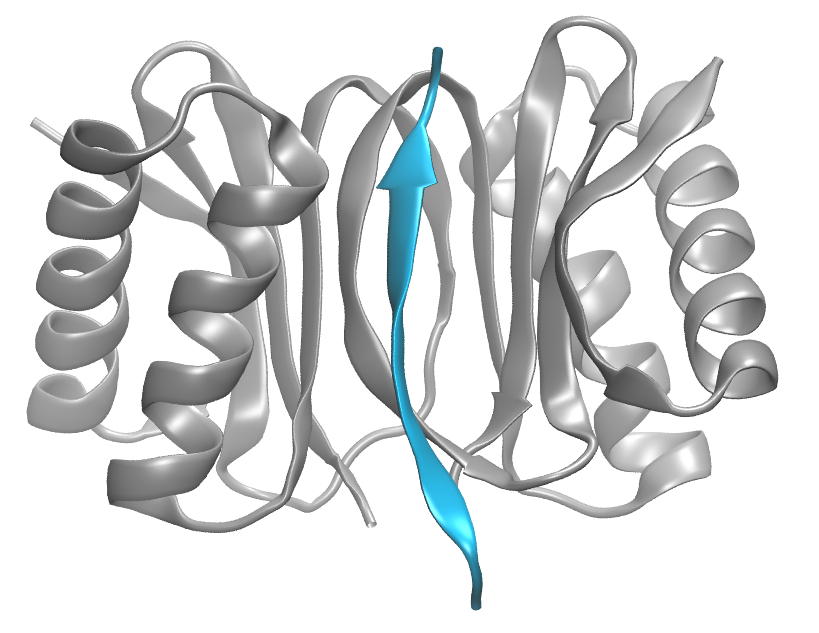


**Parassol et al., Figure S1**

Supplement: Figure S1 — DIC peptide-protein complex structure. Peptide ribbon is drawn in cyan and dynein light chain is in gray. (DOCX) [file pone.0082908.s001.docx]

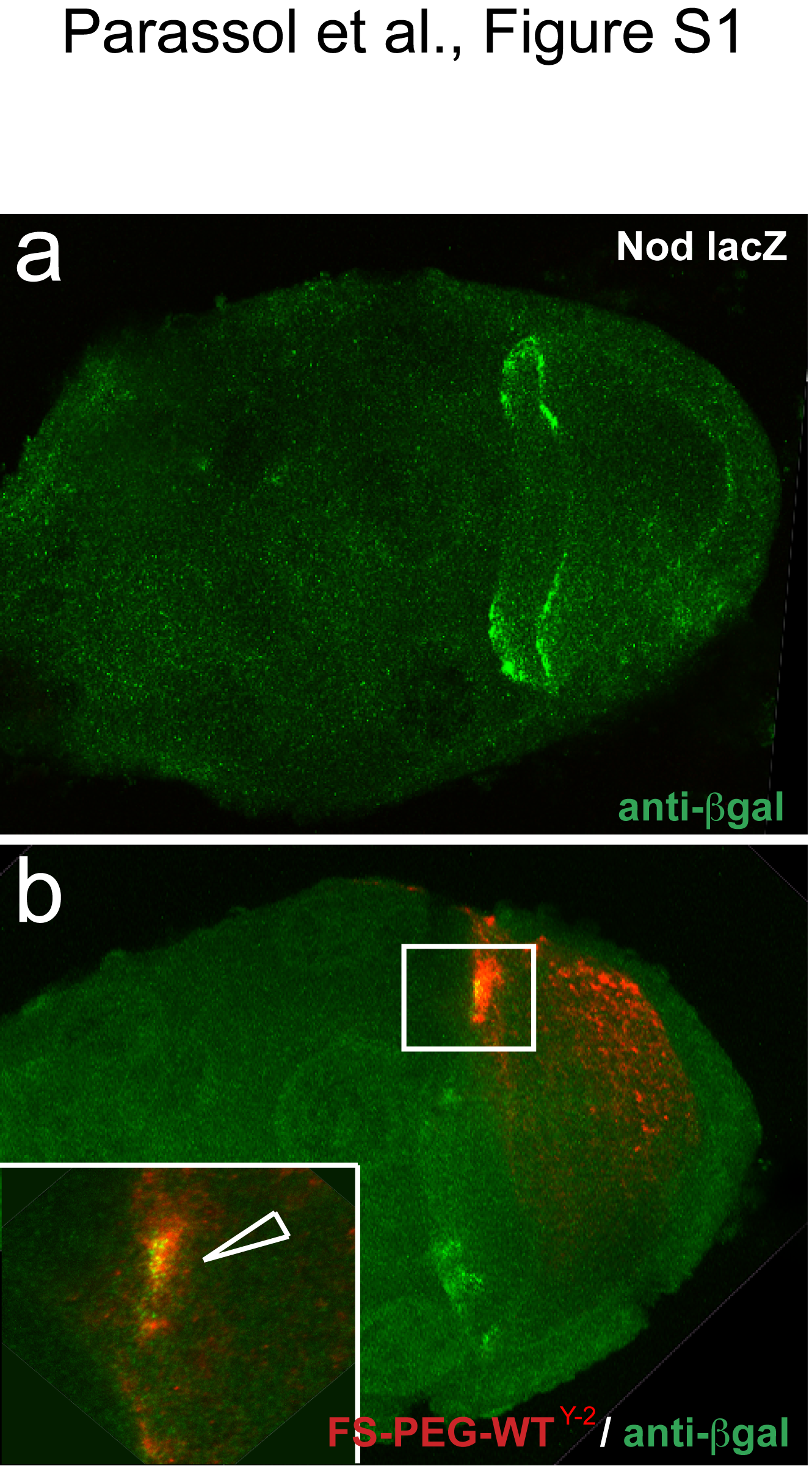

Supplement: Figure S2 — Functionalized FSs localize at the minus ends of MTs. (a) βGal staining of a Nod-LacZ oocyte showing the minus ends of MTs. (b) Localized FS-PEG-WTY-2 colocalized with βgal staining (arrow head). (TIF) [file pone.0082908.s002.tif]
