# Supplementary material for: In Vivo Characterization of Dynein-Driven nanovectors Using Drosophila Oocytes
Source: PLoS One. 2013 Dec 12;8(12):e82908. doi: 10.1371/journal.pone.0082908 (PMC3861458; doi:10.1371/journal.pone.0082908)
Supplement: Materials S1 — Molecular Modeling of Dynein peptides. (DOCX) [file pone.0082908.s003.docx]

**Materials S1: Molecular Modeling of Dynein peptides**

**Molecular modeling of dynein peptides: starting structures and docking simulations.**

Co-crystallized dynein light chain/peptide complexes containing the KXTQT motif (PDB ID = 1F95, 2P2T, 2PG1, 3E2B) were extracted from the Protein-Data Bank (PDB) and were used as initial structures for docking calculations. Water molecules were removed since peptide-protein interactions were not mediated by solvent. FoldX, a fast and quantitative computational approach to estimate free energy differences, was used to generate three-dimensional models of mutant peptides following the protocol described in Guerois *et al.* [1]. Attract, a protein-protein docking program, which has successfully predicted many receptor-ligand interfaces in recent CAPRI rounds [2]**,** was also tested to strengthen FoldX predictions. Only the ten latest residues (IVTYTKETQTP) of the best peptide binder (2PG1) were used as starting points of docking and molecular dynamics (MD) simulations (see Figure S2).

**Molecular dynamics simulations and free energy calculations.**

First principle methods based on a statistic treatment of MD simulations are required to correctly estimate thermodynamic quantities of a macromolecular system. To sample conformations of the macromolecular system, molecular dynamics simulations were carried out with the pmemd module of the AMBER10 package [3] and parm03 force-field in the isotherm-isobar thermodynamic ensemble at 300 K. Solvent was represented by a Generalized Born implicit solvent model in combination with a Langevin thermostat using a collision frequency of 1 ps to account for solvent viscosity. Translational and rotational motions were removed every 2 ps. SHAKE algorithm was used on bond involving hydrogen atoms. An infinite cut-off was applied to non-bonded van der Waals interactions and the non bonded pair list was updated every 25 steps. After having minimized the starting complex with 1000 steps of conjugate-gradient minimisation, the system was slowly heated from 100 to 300 K over a period of thermalisation of 20 ps using a distance-restraint of 10 kcal.mol^-1^.Å^-2^ on all the atoms. The resulting structure was then equilibrated without restraints during a period of 10 ns followed by a production of 50 ns. All simulations were performed with a time step of 2 ps. To avoid dependence on initial conditions, three MD simulations with different initial velocities were performed on each system (DIC, WT^Y-2^, Mut1^G-1^, Mut2^G0^, Mut3^G+1^ for a total of 5*3*50 = 750 ns simulation length. The MMGBSA method [4] is based on the analysis of configurations obtained from equilibrated MD and has been chosen since it is an efficient method to estimate free energy differences considering a series of ligands or to rank various binding mode of the same ligand [5]. Results of docking scoring and free energy calculations are summarized in Table S1. Free energy differences, estimated by the three methods above-mentioned, were combined in a consensus scoring using a simple criterion: the consensus score is positive (>0) or negative (<0) if a majority of the methods gives respectively a positive or negative free energy difference.

**Synthesis of the Peptide(P)CONH(CH_2_)_5_CONH-PEG-NH_2_ conjugates**

***1- Solid-phase synthesis of Peptide(P)CONH(CH_2_)_5_CO_2_H derivatives (steps 1-13)***

**Step 1**: grafting of the aminocaproic spacer on resin

2-Chlorotrityl chloride resin (1 g for peptide **DIC**, 650 mg for **Mut1^G-1^**, or 500 mg for **Mut2^G0^**, **Mut3^G+1^**, **WT^Y-2^**, **Egl**, and **BS69**) was swelled for 90 min in DMF (6 mL) under an inert atmosphere. After filtration and washing of the resin with DMF, *N*-Fmoc-6-aminocaproic acid (2 equiv) and *N,N*-diisopropylethylamine (DIPEA) (2 equiv) in DMF (6 mL) were added. After gentle stirring for 1 h at room temperature (rt), the resin was filtrated and washed with DMF (5 x 5 mL). This coupling reaction was repeated twice (same conditions). After capping of the resin by action of a 15:2 DMF/MeOH solution (6.8 mL), the resin was filtrated and washed with DMF (5 x 5 mL).

**Steps 2-12**: amino acid condensation and elongation

Elongation of the peptide sequence by one aminoacid residue was performed in two steps according to the Fmoc strategy, which consists first (i) into the Fmoc-deprotection, then (ii) into the condensation of the appropriate activated aminoacid. The *N*-Fmoc aminoacid building blocks used for the elongation of the peptide sequences shown in Fig. 1 were FmocProOH, FmocThr(tBu)OH, FmocGln(Trt)OH, FmocGlu(tBu)OH, FmocLys(Boc)OH, FmocTyr(tBu)OH, FmocValOH, FmocLeuOH, FmocSer(tBu)OH, FmocAlaOH, FmocAsp(tBu)OH, FmocArg(Pbf)OH, FmocHis(Trt)OH, FmocMetOH, FmocArg(Pbf)OH, FmocGlyOH. The last elongation step 12 (*N*-terminus of the peptide sequence) was performed either with an *N*-Ac- or *N*-Boc protected aminoacid, i.e. AcIleOH (or BocIleOH), AcValOH and AcProOH.

The determination of deprotection ratio (step (i)) and of the grafting ratio after each elongation step (step (ii)) was performed by titrating the Fmoc-piperidine adduct in solution with a UV spectrophotometer at 290 nm (ε = 5800) [1].

**Step (i**): the Fmoc group of the aminocaproic acid spacer or of the aminoacid was cleaved by treating the resin with 4:1 DMF/piperidine solution (4 mL) for 30 min. The resin was filtrated and successively washed with DMF (3 x 5 mL), MeOH (3 x 5 mL) and CH_2_Cl_2_ (3 x 5 mL).

**Step (ii):** the selected *N*-Fmoc amino acid (2 equiv) in DMF (6 mL) was first activated by successive addition of hydroxybenzotriazole (HOBt) (1.5 equiv) and diisopropylcarbodiimide (DIC) (1.2 equiv). Then, after stirring for 15 min at rt, the solution was added to the Fmoc-deprotected resin swelled in DMF (5 mL). The mixture was reacted for 90 min at rt, then filtrated and washed with DMF. This coupling reaction was similarly repeated three times and the resin was filtrated and successively washed with DMF (5 x 5 mL) and CH_2_Cl_2_ (5 x 5 mL). Finally, eventual unreacted amines were capped by action with a solution of acetic anhydride (0.5 M) and DIPEA (20 equiv) in DMF (4.4 mL). After 30 min reaction at rt, the resin was filtrated and successively washed with DMF (5 x 5 mL) and CH_2_Cl_2_ (5 x 5 mL).

Each intermediary peptide sequence was further checked by mass spectrometry by cleaving an aliquot of resin [4:1:1 CH_2_Cl_2_ – trifluoroethanol (TFE) - acetic acid solution (1 mL)].

**Step 13**: cleavage of the peptide sequence from resin and synthesis of **Peptide(P)CONH(CH_2_)_5_CO_2_H**

The peptide sequence was cleaved from the resin by reaction with a 4:1:1 CH_2_Cl_2_/TFE/acetic acid solution (4 mL) for 2 h at rt. These conditions allow the preservation of the lateral amino acid P (=Ac, Boc, Trt, tBu, and/or Pbf) and the *N*-terminus (Ac, Boc) protection. The resin was filtered off and washed with 1:4 TFE - CH_2_Cl_2_. The organic phase was concentrated under vacuum and the crude purified by chromatography onto Sephadex LH-60 and then silica gel chromatography (CH_2_Cl_2_/MeOH 9/1 v/v).

The isolated **Peptide(P)CONH(CH_2_)_5_CO_2_H** compounds (given hereafter as Peptide(P) code) were analyzed by electrospray ionization high-resolution mass spectrometry (ESI HRMS) in positive mode, which was performed on a LTQ/FT-Orbitrap (ThermoFisher Scientific) apparatus.

**DIC(P)**: 778 mg, yield: 51% **Egl(P)**: 300 mg, 40%; **BS69(P)**: 510 mg, 50%; **WT^Y-2^(P**): 450 mg, 55%; **Mut1^G-1^(P**): 1 g, 73%; **Mut2^G0^(P**): 400 mg, 58%; **Mut3^G+1^(P**): 450 mg, 54%. ESI HRMS of **Peptide(P)CONH(CH_2_)_5_CO_2_H** (molecular formula): observed value (theoretical value and assignments)

**DIC(P)** (Ac form) (C_113_H_176_N_14_O_24_): 2136.2935 (2136.2880, [M + Na]^+^)

**Egl(P)** (Ac form) (C_100_H_158_N_14_O_23_): 2136.2935 (2136.2880, [M + Na]^+^)

**BS69(P)** (Ac form) (C_142_H_199_N_21_O_25_S_3_): 1348.2168 (1348.2133, [M + 2H]^2+^)

**WT^Y-2^(P)** (Boc form) (C_120_H_184_N_14_O_24_): 2228.3499 (2228.3506, [M + Na]^+^)

**Mut1^G-1^(P)** (Boc form) (C_110_H_170_N_14_O_24_): 1036.6321 (1036.6335, [M + 2H]^2+^)

**Mut2^G0^(P)** (Ac form) (C_91_H_157_N_13_O_23_): 1801.1597 (1801.1594, [M + H]^+^)

**Mut3^G+1^(P)** (Ac form) (C_107_H_164_N_14_O_23_): 1007.6155 (1007.6125, [M + 2H]^2+^)

***2- Liquid-phase synthesis of* Peptide(P)CONH(CH_2_)_5_CONH-PEG-NH_2_**

HOBt (1.5 equiv) and DIC (1.2 equiv) were successively added to a solution of the above **Peptide(P)CONH(CH_2_)_5_CO_2_H** (1.2 equiv) in CH_2_Cl_2_ (3 mL). After stirring for 12 h, DIPEA (2 equiv) and **NH_2_-PEG-NHFmoc** [= NH_2_-CH_2_-CH_2_-(O-CH_2_CH_2_)_n_-NHFmoc (n ≈ 110; 1 equiv] were added to the solution and stirring at rt was continued for 24 h. Then, the mixture was concentrated under vacuum. The crude product was purified by size exclusion chromatography (Sephadex LH-60, 95:5 CH_2_Cl_2_ - MeOH) giving **Peptide(P)NH-PEG-NHFmoc** as a white solid. This solid was then poured into 3 mL of 1:4 piperidine – DMF solution at rt for 30 min. The resulting mixture was then purified by size exclusion chromatography (Sephadex LH-60, methanol) giving quantitatively **Peptide(P)NH-PEG-NH_2_**.

The isolated **Peptide(P)CONH(CH_2_)_5_CONH-PEG-NH_2_** compounds were analyzed by ESI HRMS; which are given hereafter as Peptide(P) code) (molecular formula): observed value (theoretical value and assignments).

**DIC(P)** (Boc form) (C_336_H_624_N_16_O_133_ for n = 109): 1182.3530 (1182.3691, [M+4H+2K]^6+^)

**DIC(P)** (Ac form) (C_335_H_622_N_16_O_133_ for n = 110): 1197.3564 (1197.3506, [M+3Na+3K]^6+^)

**Egl(P)** (Ac form) (C_338_H_636_N_16_O_140_ for n = 118): 1219.6999 (1219.6974, [M + 2H + 4K]^6+^)

**BS69(P)** (Ac form) (C_370_H_657_N_23_O_137_S_3_ for n = 113): 1293.5868 (1293.5737, [M + 4H + 2Na]^6+^)

**WT^Y-2^(P)** (Boc form) (C_342_H_630_N_16_O_133_ for n = 110): 1419.0364 (1419.0683, [M + 5H]^5+^)

**Mut1^G-1^(P)** (Boc form) (C_338_H_628_N_16_O_136_ for n = 113): 1188.6909 (1188.7124, [M + 5H +K]^6+^)

**Mut2^G0^(P)** (Ac form) (C_319_H_615_N_15_O_135_ for n = 113): 1143.5002 (1143.5291, [M + 5H +K]^6+^)

**Mut3^G+1^(P)** (Ac form) (C_335_H_622_N_16_O_135_ for n = 113): 1179.0175 (1179.0388, [M + 5H +K]^6+^)

**References**

1. Guerois R, Nielsen JE, Serrano L (2002) *J. Mol. Biol.* **320:** 369-87

2. Fiorucci S, Zacharias M (2010) *Proteins* **78:** 3131-3139

3. Case DA, Darden TA, Cheatham TE, Simmerling CL, Wang J et al. (2008) *AMBER 10, University of California, San Francisco.*

4. Kollman PA, Massova I, Reyes C, Kuhn B, Huo S et al. (2000) *Acc. Chem. Res.* **33:** 889-897

5. Fiorucci S, Antonczak S, Golebiowski J, (2010) Chap.XI in *Protein-protein complexes: Analysis, modeling and drug design*, M. Zacharias, Imperial Press.
