# Supplementary material for: In Vivo Characterization of Dynein-Driven nanovectors Using Drosophila Oocytes
Source: PLoS One. 2013 Dec 12;8(12):e82908. doi: 10.1371/journal.pone.0082908 (PMC3861458; doi:10.1371/journal.pone.0082908)
Supplement: Table S1 — Binding free energy differences of mutant sequences related to DIC peptide according to various computational methods. (DOCX) [file pone.0082908.s014.docx]

**Parassol et al., Table S1**

|  |  | **ΔΔG^*^ (kcal.mol^-1^)** | | | |
| --- | --- | --- | --- | --- | --- |
| **Name** | **Sequence** | **FoldX** | **Attract** | **MMGBSA** | **Consensus score** |
| DIC | IVTYTKETQTP | - | - | - | - |
| WT^Y-2^ | IVTYTKYTQTP | 0.01 | -2.54 | -7.82 | <0 |
| Mut1^G-1^ | IVTYTKEGQTP | 0.1 | 1.38 | 27.57 | >0 |
| Mut2^G0^ | IVTYTKETGTP | 0.82 | 0.86 | 31.01 | >0 |
| Mut3^G+1^ | IVTYTKETQGP | 0.92 | 0.71 | 48.09 | >0 |

^*^ΔΔG_cpx =_ ΔG_cpx_MUT -_ ΔG_cpx DIC_
